# *Craterispermum capitatum* and *C. gabonicum* (Rubiaceae): two new species from the Lower Guinean and Congolian Domains

**DOI:** 10.3897/phytokeys.83.13623

**Published:** 2017-07-21

**Authors:** Herman Taedoumg, Bonaventure Sonke, Perla Hamon, Petra De Block

**Affiliations:** 1 Bioversity International, P.O. Box 2008 Messa, Yaoundé, Cameroon; 2 Plant Systematic and Ecology Lab, Higher Teacher’s Training College, University of Yaoundé I, PO Box 047, Yaoundé, Cameroon; 3 Institut de Recherche pour le Développement, UMR DIADE, 911 avenue Agropolis, BP 64501, 34394 Montpellier CEDEX 5, France; 4 Botanic Garden Meise, Nieuwelaan 38, 1860 Meise, Belgium

**Keywords:** Rubiaceae, *Craterispermum*, *C. capitatum*, *C. gabonicum*, dimorphic inflorescences and flowers, dioecy, heterostyly, Cameroon, Gabon, Nigeria, Congo, DR Congo

## Abstract

*Craterispermum capitatum* and *C. gabonicum*, two new species of Rubiaceae, are described from the Lower Guinea and Congolian Domains. Detailed descriptions and distribution maps are provided for each species, their conservation status is assessed and their taxonomic affinities are discussed. *Craterispermum gabonicum* is unique within the genus because of the strong dimorphism in brevistylous and longistylous flowers and inflorescences. We hypothesize that this species shows some form of dioecy. The distribution of *C. capitatum* shows a wide disjunction: the species is present in the Lower Guinean and Congolian Domains but absent from Gabon and South Cameroon. An identification key for the *Craterispermum* species present in the Lower Guinean and Congolian Domains is given.

## Abstract (fr)

*Craterispermum capitatum* et *C. gabonicum*, deux nouvelles espèces de la famille des Rubiaceae, des Domaines Bas Guinéen et Congolais sont décrites. Des descriptions détaillées et des cartes de distribution sont données pour chacune des espèces, leur statut de conservation est évalué et leurs affinités taxonomiques discutée. *Craterispermum gabonicum* est unique dans le genre en raison d’un dimorphisme avéré entre les fleurs, mais aussi les inflorescences brévistyles et longistyles. L’hypothèse de l’existence d’une tendance vers la dioécie chez cette espèce est émise. La distribution *C. capitatum* présente une importante disjonction: l’espèce est présente dans les Domaines Bas Guinéen et Congolais, mais absente au Gabon et au sud du Cameroun. Une clé d’identification pour les espèces de *Craterispermum* présentes dans les Domaines Bas Guinéen et Congolais est donnée.

## Introduction

The genus *Craterispermum* Benth. (Rubiaceae, subfamily Rubioideae) is distributed in tropical Africa, Madagascar and the Seychelles (Robbrecht 1988, Taedoumg et al. 2011, Taedoumg and Hamon 2013; De Block and Randriamboavonjy 2015). *Craterispermum* species are shrubs or small trees with axillary or supra-axillary inflorescences, paired at the nodes and often condensed. The flowers are few to many per inflorescence, small, heterostylous and white. The ovary is bilocular with a single, apically attached, pendulous ovule in each locule. One ovule aborts and the fleshy fruit contains a single seed, shaped like an asymmetrical shallow or deep bowl. The seed has a peculiar, discontinuous seed coat, comprised of isolated cells with ring-like thickenings (Igersheim 1992). Raphides are present in all plant tissues in the genus. *Craterispermum* species have been shown to accumulate aluminium in leaves and stem tissue (Jansen et al. 2000); the leaves dry pale yellow or green, which is typical for aluminium accumulating species.

According to Anderson (1973), there are probably more heterostylous species in the Rubiaceae than in all other angiosperm families put together. In the genus *Craterispermum* heterostyly was often overlooked and plants with different floral morphs were sometimes described as separate species. For example, the type of *C. congolanum* De Wild. & Th. Dur. is just the brevistylous morph of *C. angustifolium* De Wild. & Th. Dur. De Wildeman, 1924 was one of the first to notice that some described species were just different morphs of heterostylous species. In *Craterispermum* and in other heterostylous Rubiaceae species (e.g. *Psychotria* L.), thrum flowers characteristically have included styles and exserted anthers; pin flowers have exserted styles and included anthers (complete heterostyly) (Robbrecht 1988).

*Craterispermum* is easily recognized at the genus level, but many of the species look similar and identification at the species level is difficult (Verdcourt 1973, Taedoumg et al. 2011). Herbarium material of *Craterispermum* is often poor, generally carrying only residual inflorescences. Because of the compact structure of the inflorescences, flowers and fruits fall easily during collecting, pressing, drying and mounting. Moreover, flowers are short-lived and ripe fruits do not remain on the plant for long (Taedoumg et al. 2011). The above-mentioned reasons make *Craterispermum* species challenging to describe.

The examination of the available herbarium material allowed us to highlight the existence of several new species. Hitherto, we have described five species from continental Africa (Taedoumg et al. 2011; Taedoumg and Hamon 2013). The present paper describes two further species from Cameroon, Gabon, Nigeria, Congo and the Democratic Republic of Congo. An identification key for the *Craterispermum* species present in the Lower Guinean and Congolian Domains is also given.

## Methods

Herbarium material of the following institutions was studied: BR, BRLU, G, K, MO, P, WAG and YA. Descriptive terminology follows Robbrecht (1988) and Anonymous (1962). Phytogeographical terminology follows White (1979). Measurements and other given details are based on the study of herbarium specimens, using a Leica MZ95 stereomicroscope, and data derived from field notes. In the descriptions and key, inflorescence size does not include the corollas, and given colours (except flower colour) are for dried material. Inflorescences are described as uniflorous (one flower only), pauciflorous (2 to 9 flowers) or multiflorous (10 to up to 50 flowers). Flowering and fruiting periods are given as cited on the collector’s labels.

Specimens are cited per country, alphabetically by first collector. All cited specimens have been seen. Coordinates are given to minute-level for each specimen. In the specimen citations “sl” and “sd” indicate that collection locality and date, respectively, are missing on the herbarium label. The conservation status was assessed by applying the IUCN Red List Category criteria (IUCN 2012) using the Geospatial Conservation Assessment Tools in GeoCAT (Bachman et al. 2011). The key covers the countries Nigeria, Cameroon, Equatorial Guinea, Gabon, Congo and D.R.Congo.

## Taxonomic treatment

### Craterispermum capitatum

Taxon classificationPlantaeORDOFAMILIA

Taedoumg & De Block sp. nov.

urn:lsid:ipni.org:names:77164218-1

Figs 1 , 2

#### Diagnosis.

Resembling *C. robbrechtianum* Taedoumg & Sonké, 2011 by the coriaceous leaves, the obscure intersecondary venation especially in fresh condition and the ovoid shape of the young fruits, but differing from this species by the capitate structure of the inflorescence (vs branched and subcapitate in *C. robbrechtianum*), the ovoid shape of its fruits at maturity (vs asymmetrically subglobular), the granular texture of the young branches (vs smooth), and the leaf blades generally glossy above in dry condition (vs dull).

**Figure 1.**
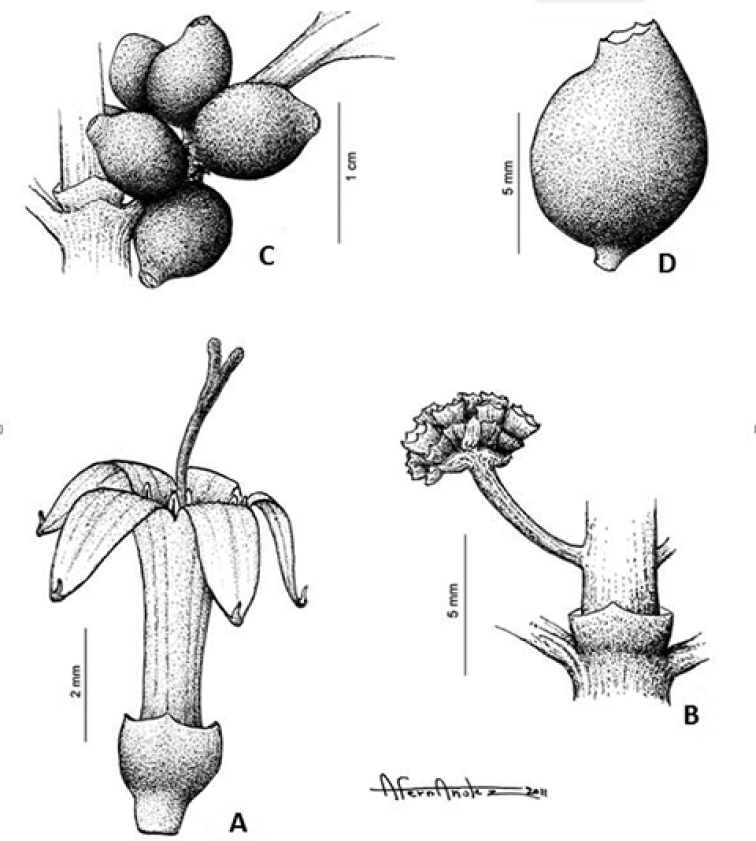
*Craterispermum capitatum*. **A** Longistylous flower **B** Node with stipules and inflorescences (only one shown) **C** Node with infrutescence with young fruits **D** Young fruit. **A** from Trochain 8306 (P) **B** from Richards 3382 (MO) **C, D** from Louis 9162 (BR). Drawn by Antonio Fernandez.

#### Type.

DEMOCRATIC REPUBLIC OF THE CONGO. Yangambi, à 6,5 km au NW du Poste, 0°46'N, 24°27'E, 470 m, 6 March 1937 (fr), *J.L.P. Louis 3440* (holotype BR [0000008055132])

Shrub or treelet up to 8 m tall; all vegetative and reproductive parts glabrous externally. Stems pale grey, ca. 10 cm in diameter; young branches grayish to brownish, often granular in outlook, often with more or less quadrangular sections, generally canaliculate near the final nodes. Stipules persistent, sheath 1–3 mm long, truncate to subtruncate or rarely with short awn ca. 0.5 mm long. Leaves petiolate; petioles canaliculate, 10–17 mm long; leaf blades narrowly elliptic or narrowly oblong to obovate, 8–20.5 × 3–7 cm, coriaceous, green, greenish brown or gold green and glossy above, paler green below; base cuneate; apex acuminate, acumen 7–10 mm long; margins somewhat revolute; midrib prominent below; secondary nerves 8–9 pairs, somewhat prominent on both surfaces, intersecondary venation obscure on both surfaces, almost invisible in fresh condition. Inflorescences supra-axillary, borne 1–5 mm above the nodes, erect, capitate, 4–9 × 1.8–4 mm, pauciflorous to multiflorus; peduncle flattened, 1.5–7 mm long; bracts and bracteoles broadly triangular, 1–1.6 mm and ca. 0.8 mm long respectively, apex obtuse or truncate, margins sometimes bearing sparse colleters. Flowers presumed heterostylous (but only longistylous morph known), 5-merous, sessile. Longistylous flowers: Calyx greenish white; tube 0.5–0.7 mm long, subtruncate or with short, obtuse teeth ca. 0.3 mm long, margins sometimes sparsely bearing colleters. Corolla white; tube narrowly cylindrical, 4–5 mm long, sparsely to densely pubescent in the throat and upper quarter inside; lobes ca. 2.5 mm long, glabrous or sparsely pubescent in the basal half inside, tips acute. Stamens inserted below the level of the throat, only apices exserted from corolla tube at anthesis; anthers ca. 1.1 mm long, white; filaments ca. 0.2 mm long. Ovary ca. 1.1 mm long, greenish white. Style and stigma exserted from the corolla tube at anthesis, ca. 6 mm long, glabrous; stigma bilobed, stigmatic lobes ca. 1.4 mm long. Infrutescences carrying (2–)4–10 fruits. Fruits sessile, ovoid, 8–10 mm diam., successively green, whitish green and dark violet at maturity.

#### Taxonomic affinities.

This species is morphologically close to *C. robbrechtianum* because of its coriaceous leaves, its obscure intersecondary venation especially in fresh material, the length of its peduncles and the shape of its young fruits. However, it differs from this species by the capitate structure of its inflorescence (vs branched and subcapitate in *C. robbrechtianum*), the ovoid shape of its fruits at maturity (vs asymetrically subglobular in *C. robbrechtianum*), the granular texture of the young branches (vs smooth in *C. robbrechtianum*), and its leaf blades generally glossy above in dry condition (vs dull in *C. robbrechtianum*). In addition, fruiting herbarium specimens tend to retain more fruits [(2–)4–10 fruits] (vs ca. 1–2 fruits in *C. robbrechtianum*).

The specimens of *C. capitatum* studied were almost all previously identified as *C. cerinanthum* Hiern. But this species clearly differs from *C. capitatum* by its relatively long pedunculate, branched and lax inflorescences.

#### Phenology.

Flowers: March - May (Nigeria), July (Cameroon); October (Republic of the Congo); Fruits: March - May, September - December (Democratic Republic of the Congo); April (Nigeria).

#### Distribution and habitat.

**Figure 2.**
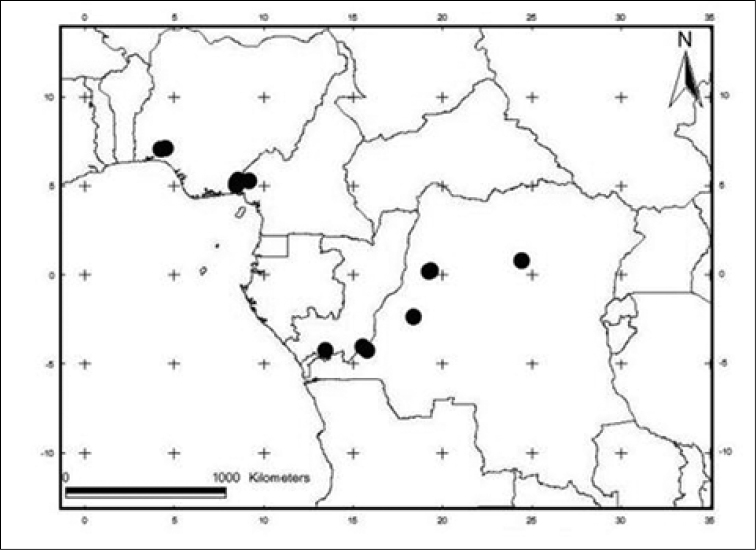
Distribution map of *Craterispermum capitatum*.

*Craterispermum capitatum* is known from Western Cameroon, the Democratic Republic of the Congo, South-Eastern Nigeria and the Republic of the Congo. It grows in semi-deciduous primary and secondary forest between 0 and 470 m elevation (Fig. 2).

#### Vernacular name and uses.

Democratic Republic of the Congo - Botele bo lokonda (Turumbu); Djeli na Kupi (-). Leaves are used as fetish to avoid panthers.

#### Preliminary conservation status.

IUCN status:—Vulnerable: VU B2b(iii). The extent of occurrence (EOO) of *C. capitatum* is 1,134.21 km², and its area of occupancy (AOO) is 52 km² using a cell width of 2 km. The species is distributed in 7 subpopulations, 2 of which are located in protected areas: the Omo-Oluwa-Shasha Forest Reserve located in Ondo State in Nigeria and the Korup National Park in Cameroon and Cross River National Park in Nigeria, which are in fact contiguous. Habitat loss outside the protected areas is a serious threat for *C. capitatum*, but loss of forest is also documented for the Omo-Oluwa-Shasha Forest Reserve and the Cross River National Park in Nigeria (Ite 1997; Adedeji and Adeofun 2014).

Field study is required to fully assess the AOO of *C. capitatum* and, given the fact that the Democratic Republic of the Congo is not well collected, the number of locations for the species is likely to increase.

#### Etymology.

The name of the species was chosen because of the capitate structure of its inflorescences.

#### Critical notes.

The distribution of *C. capitatum* is atypical because of its absence in the South of Cameroon and in Gabon. While rare, several other Rubiaceae species show distribution patterns with a similar macro-disjunction, notably *Hymenocoleus rotundifolius* (A. Chev. ex Hepper) Robbr. (Robbrecht 1996) and *Ixora brachypoda* DC. (De Block 1998). The reason of this atypical distribution is not yet clearly determined, but in this case, it is probable that the continuous humid forest in southern Cameroon and Gabon is not an ideal habitat for *C. capitatum*,which occurs mostly in more semideciduous forests.

#### Additional specimens examined

**(paratypes). CAMEROON**: NE corner of Korup National Park, near Baro Village, 5°16'N, 9°11'E, 200 m, 24 March 1984 (fl), *D.W. Thomas 3358* (MO, WAG). **REPUBLIC OF THE CONGO**: M’Boku-COFORIC, Forêt du Mayumbe, 4°15'S, 13°29'E, 8 October 1950 (fl), *J. Trochain 8306* (P). **NIGERIA**: South-West, Shasha Forest Reserve, 1/4 mile SW of Osho enclave, 7°5'N, 4°30'E, 1 April 1946 (fl bud), *A.D.P. Jones FHI 17233* (BM, K, P); Omo and Shara Forest Reserve, about 1/2 mile SW of Osho enclave, site of E.B.3L., 7°0'N, 4°15'E, 3 April 1946 (fr), *A.P.D. Jones & C.F. Onochie FHI 17352* (K, P); Omo Forest Reserve, 3 km S of Aberu, 10 km S of Omo Sawmill, 7°0'N, 4°15'E, 14 May 1980 (fl), *E. Pilz 2455* (MO); Ogun, Omo Forest Reserve, 3 km S of Aberu, 10 km S of Omo Sawmill, 7°0'N, 4°15'E, 17 May 1980 (fl), *E. Pilz 2530* (MO, WAG); Ijebu Province, Shasha Forest Reserve, 7°5'N, 4°30'E, 4 March 1935 (fl), *P.W. Richards 3192* (BM, MO); Ijebu Province, Shasha Forest Reserve, 7°5'N, 4°30'E, 22 April 1935 (fr), *P.W. Richards 3382*, (BM, MO); Ijebu Province, Shasha Forest Reserve, 7°5'N, 4°30'E, 12 April 1935 (fr), *R. Ross 210* (BM, MO); Oban, 5°19'N, 8°34'E, 1911 (fr), *P.A. Talbot 208* (BM); South Eastern State, Ekinta River Forest Reserve, about 20 km ENE of Calabar, 5°0'N, 8°30'E, 1 April 1971 (fr), *P.P.C. Van Meer 1113A* (WAG); South Eastern State, Ekinta River Forest Reserve, about 20 km ENE of Calabar, 5°0'N, 8°30'E, 2 April 1971 (fl, fr), *P.P.C. Van Meer 1124* (WAG); South Eastern State, Oban Group Forest Reserve, West block, between pillar 59 and 60, Kwa River, 5°9'N, 8°28'E, 150 m, 14 April 1971 (fr), *P.P.C. Van Meer 1450* (WAG). **DEMOCRATIC REPUBLIC OF THE CONGO**: route Mabana km 3, terr. Maluku, 4°3'S, 15°33'E, 27 November 1970 (fr), *H. Breyne 982* (BR); Sualempu, 12 km de Bita, 4°16'S, 15°48'E, 24 March 1971 (fr), *H. Breyne 2125* (BR); sl, 1921 (fr), *J. Claessens 644* (BR); Bokolongo-Djoa, terr. Bolomba, 0°12'N, 19°21'E, 27 February 1958 (fr), *C. Evrard 3569* (BR); Djoa (territoire Bolomba), 0°8'N, 19°16'E, 17 May 1958 (fr), *C. Evrard* 4075 (BR); Djoa, terr. Bolomba, 0°8'N, 19°16'E, 15 October 1958 (fr), *C. Evrard 5014* (BR); Bankaie, terr. Inongo 2°22'S, 18°25'E, 9 September 1953 (fr), *G. Gilbert 14772* (BR); Yangambi, plateau de la Luweo, 0°46'N, 24°27'E, 470 m, 30 April 1938 (fr), *J. Louis 9162* (BR).

### Craterispermum gabonicum

Taxon classificationPlantaeORDOFAMILIA

Taedoumg & De Block sp. nov.

urn:lsid:ipni.org:names:77164219-1

Figs 3 , 4

#### Diagnosis.

Resembling *C. ledermannii* K. Krause, 1912 because of the large leaves and the often robust peduncles, but differing from this species by the subcapitate inflorescences (vs branched in *C. ledermannii*), the notable dimorphism between flowers and inflorescences of the different flower morphs, the secondary nerves clearly ascending and forming acute angles with the midrib (vs secondary nerves more or less perpendicular to the midrib).

**Figure 3.**
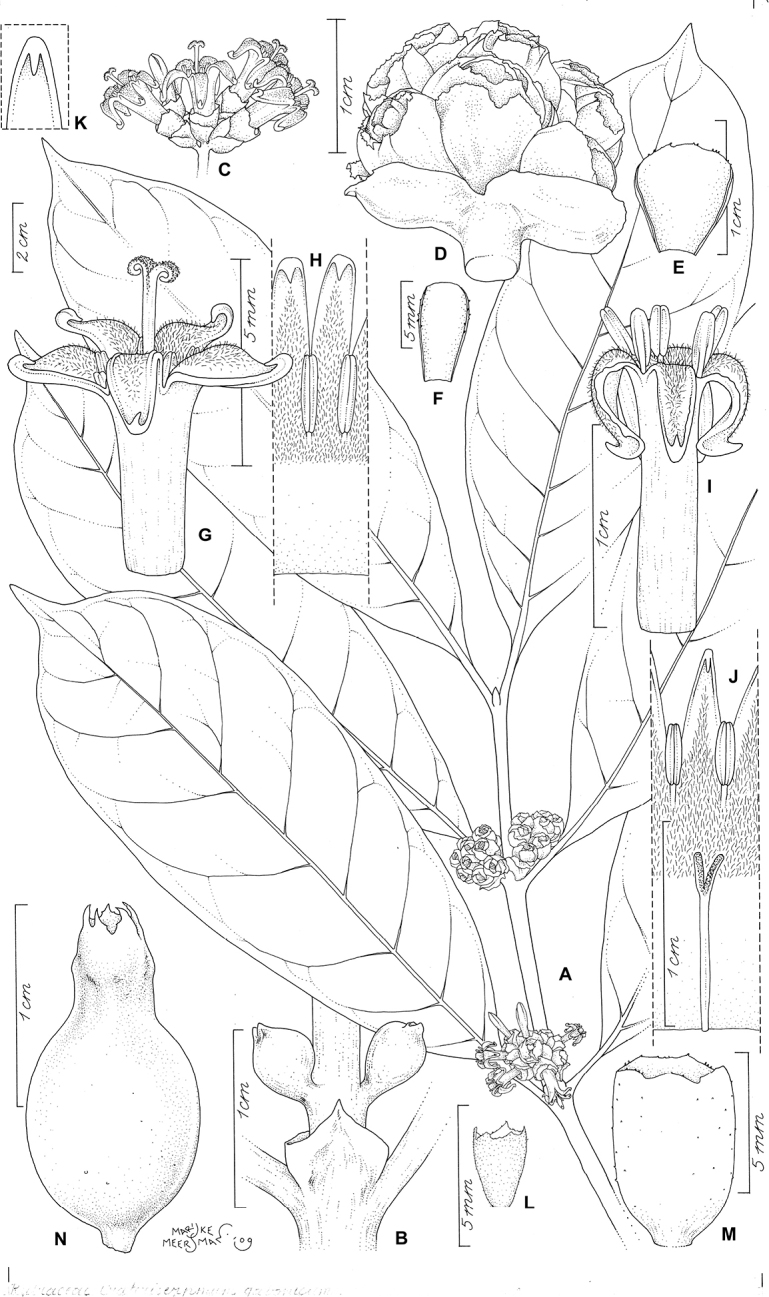
*Craterispermum gabonicum*. **A** Flowering branch (brevistylous morph) **B** Node with stipules and young inflorescences **C** Inflorescence (longistylous morph) **D** Inflorescence (corollas fallen) (brevistylous morph) **E** Bracteole (brevistylous morph) **F** Bracteole (longistylous morph) **G** Corolla (longistylous morph) **H** Longitudinal section of corolla (longistylous morph) **I** Corolla (brevistylous morph) **J** Longitudinal section of corolla (brevistylous morph) **K** Tip of corolla lobe showing subapical spike-like protuberance **L** Calyx (longistylous morph) **M** Calyx (brevistylous morph) **N** Immature fruit. **A–B, D–E, G–H, M** from *Wieringa 1611* (WAG), **C, F, I–K, L** from *Issembe 244* (WAG), N from *Breteler 10979* (WAG). Drawn by Marijke Meersman.

#### Type.

GABON. Ogooué-Maritime: Rabi-Kounga, ca. 4 km N of Shell-camp, 1°55'S, 9°52'E, 19 September 1992 (fl), *J.J. Wieringa & J.B. Epoma 1611* (holotype WAG [WAG0233599], isotype WAG [WAG0233600]).

Shrub or treelet, 1.5–9 m tall; all vegetative and reproductive parts glabrous externally. Stems brownish, ca. 8 cm in diameter; young branches greenish or grayish, somewhat granular in outlook. Stipules persistent, sheath 2–7 mm long, keeled, subtruncate or with short awn <1 mm long. Leaves petiolate; petioles canaliculate, 7–19 mm long; leaf blades narrowly elliptic or narrowly obovate, more rarely elliptic or obovate, 10.5–24 × 4.4–7.2 cm, coriaceous, brownish or yellowish green and generally dull above, paler green or brown below; base cuneate; apex acuminate, acumen 8–16 mm long; margins not revolute; midrib prominent below; secondary nerves 6–9 pairs, clearly ascending and forming acute angles with midrib, obscure on both surfaces; intersecondary venation moderately prominent above, obscure on lower surface, obscure to almost invisible on both surfaces in fresh condition. Inflorescences axillary to supra-axillary, borne up to 7.5 mm above the nodes, erect, subcapitate to capitate, 4–19 × 5–20 mm; peduncle subcylindrical to flattened (up to 3.5 mm wide), robust, 1.5–4.5(–7) mm long. Inflorescences completely covered by imbricate outer bracts when young, medium green to greenish white (somewhat resembling an immature fruit); brevistylous and longistylous inflorescences dimorphous: brevistylous inflorescences very congested, 11.5–19 × 7.5–20 mm, pauciflorous to multiflorous; bracts and bracteoles broadly triangular or ovate, 6–8 × 5–8 mm and 6 × 3 mm respectively with rounded or rarely subtruncate apex and margins sometimes bearing sparse colleters; longistylous inflorescences less congested, 4–7 × 5.2–9.1 mm, pauciflorous; bracts and bracteoles broadly triangular, ca. 2 × 1.5–2 mm and 0.7–2 × 1–2 mm respectively with rounded or truncate apex and margins sometimes bearing sparse colleters. Flowers heterostylous, 5-merous, sessile; ovary and calyx pale green or whitish, somewhat violet tinged; corolla narrowly cylindrical, white; anthers and filaments white or whitish violet. Brevistylous flowers: Calyx with tube (1.5–)3–5 mm long, sometimes bearing sparse colleters at the base inside; lobes (0.3–0.6)–1.5 mm long, apex acute, obtuse or rounded, margins sometimes bearing sparse colleters. Corolla with tube 6–12 mm long, pubescent in the throat and upper half inside; lobes 3–6 mm long, covered with short hairs at the base inside, tips acute. Stamens inserted ca. 2 mm below the level of the throat, completely exserted from or only bases included in corolla tube at anthesis; anthers 1.1–3 mm long; filaments 1–2.5 mm long. Ovary 0.5–1.2 mm long. Style and stigma included in corolla tube at anthesis, 4–7.5 mm long; stigma bilobed, stigmatic lobes 1.5–2 mm long. Longistylous flowers: Calyx with tube 1–1.5 mm long; lobes triangular, 0.5–0.7 mm long sometimes bearing sparse colleters at the base inside. Corolla with tube 4–5 mm long, pubescent in the throat and upper third inside; lobes 3.5–4 mm long, densely pubescent at the base inside, tips acute. Stamens inserted ca. 2 mm below the level of the throat; completely included in the corolla tube or only the apices exserted at anthesis; anthers 1.8–2 mm long; filaments <0.5 mm long. Ovary ca. 1.2 mm long. Style and stigma exserted from the corolla tube at anthesis, ca. 7.5 mm long; stigma bilobed, stigmatic lobes ca. 1.5 mm long. Fruits sessile, urceolate, ca. 16 × 8 mm, colour at maturity unknown.

#### Taxonomic affinities.

This species is atypical in the genus because of the flower and inflorescence dimorphism. However, it somewhat resembles *C. ledermannii* because of the large leaf blades and the often robust peduncles. It differs from this species by its subcapitate inflorescences (vs mostly branched in *C. ledermannii*), its secondary nerves clearly ascending and forming acute angles with the midrib (vs more or less perpendicular to the midrib in *C. ledermannii*) and by the fact that its inflorescences are completely covered by imbricate outer bracts when young (somewhat resembling an immature fruit) (vs never completely covered in *C. ledermannii*).

#### Phenology.

Flowers: March-May and August-November. Fruits: February, April and August-December.

#### Distribution and habitat.

**Figure 4.**
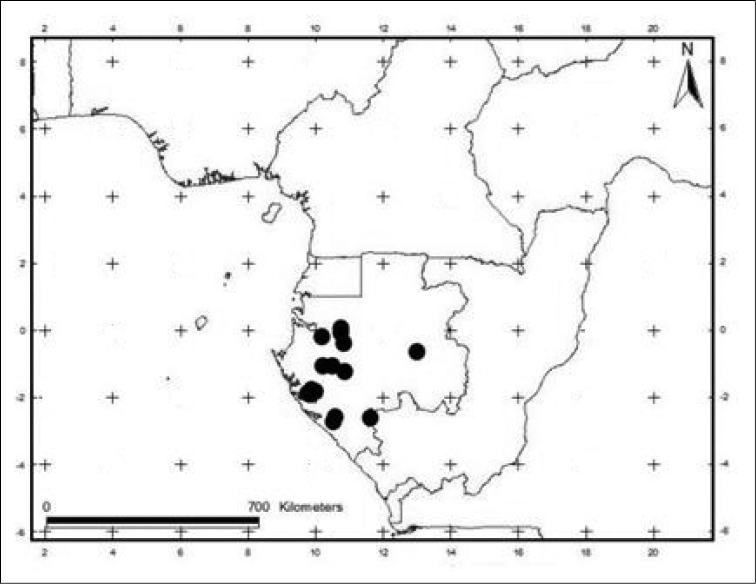
Distribution map of *Craterispermum gabonicum*.

*Craterispermum gabonicum* is endemic to Gabon. It grows in primary forest but also in humid secondary forest between 150 and 400 m elevation (Fig. 4).

#### Vernacular names and uses.

Unknown.

#### Preliminary conservation status.

IUCN status:—Vulnerable: VU B2(iii). The extent of occurrence (EOO) of *C. gabonicum* is 80,847.46 km², and its area of occupancy (AOO) is 60 km² using a cell width of 2 km. The species is distributed in 7 or 8 subpopulations, 2 of which are located in protected areas: in Wonga Wongué Forest Reserve in Ogooué-Maritime Division and on the edge of the Loango National Park in West Gabon. The Wonga Wongué Forest Reserve is subject, these last years, to a very strong pressure from uncontrolled anthropomorphic activities (illegal exploitation of the resources as well as degradation of the ecosystems as a result of oil exploitation). Habitat loss outside and inside the protected areas is a serious threat for *C. gabonicum*.

#### Etymology.

This species is named after the country to which it is currently endemic.

#### Critical notes.

Within this species flowers and inflorescences are very variable in shape and size, more so than in other species of the genus. This variability seems to be *a priori* correlated with the heterostyly, which is present in all species of the genus. However, *C. gabonicum* does not only show the reciprocal stigma and anther position and the pollen dimorphism typical for heterostylous species and present in all continental African *Craterispermum*, but a further dimorphism occurs at flower and at inflorescence level. The corolla tube of brevistylous flowers is longer and wider than that of longistylous flowers (6–12 mm × ca. 3 vs 4–5 × 1.5–2 mm) (Fig. 3 H–I, K–L). Brevistylous flowers generally also have longer and wider calyx tubes than longistylous flowers [(1.5–)3–5 mm vs 1–2 mm long) (Fig. 3J–G). Except for the length of the filaments, possible size differences in anthers and stigmatic lobes could not conclusively be observed. In regard to the inflorescences, those with brevistylous flowers comprise more flowers than those with longistylous ones (multiflorous vs pauciflorous, respectively)(Fig. 3C–D). Furthermore, bracts and bracteoles are larger in inflorescences with brevistylous flowers than in inflorescences with longistylous flowers (6–8 × 5–8 mm and ca. 6 × 3 mm respectively vs ca. 2 × 1.5–2 mm and 0.7–2 × 1–2 mm) (Fig. 3E–F).

These differences in size are unknown in heterostylous species but are typical for certain dioecious ones (Pailler et al. 1998; Lantz and Bremer 2004; Mouly and Achille 2007). In several plant groups dioecy has been shown to have evolved from heterostyly, with the functionally male flowers derived from the brevistylous and the functionally female flowers from the longistylous morphs (Beach and Bawa 1980). This is also the case for certain Rubiaceae species, such as *Chassalia corallioides* (Cordem.) Verdc. (Pailler et al. 1998) and *Mussaenda parviflora* Miq. (Naiki and Kato 1999). The size difference in flowers and inflorescences observed in *C. gabonicum* is also reported from certain dioecious species. Fewer flowers per inflorescence are found in individuals with female flowers than in individuals with male flowers in dioecious species of the tribe Vanguerieae (Lantz and Bremer 2004; Mouly and Achille 2007). Also, in certain dioecious species, such as *Chassalia corallioides* (Pailler et al. 1998) male flowers have longer corolla tubes than female flowers. We therefore suggest that a trend towards functional dioecy could be the explanation for the dimorphism in flowers and inflorescences in *C. gabonicum*, with the flowers being morphologically heterostylous but functionally dioecious or evolving towards this condition.

It is very difficult to verify this hypothesis without field studies, especially since mature flowers of both morphs and mature fruits are rare on the available herbarium material and no fixed flower material was available for detailed morphological and anatomical studies. With hardly any fruit set, it is impossible to know whether only one (brevistylous) or both morphs set fruit. Furthermore, both morphs produce viable pollen (based on morphological characters) although anthers are somewhat larger and pollen more abundant in the brevistylous morph. While the calyx tube is much longer in the brevistylous morph, this is not the case for the ovaries, which rather are somewhat reduced. All ovaries of brevistylous flowers contained ovules, but these too seemed somewhat reduced in size. Because of the lack of available plant material with mature flowers and fruits, it is impossible to demonstrate with certainty the hypothesis stated here that dioecy in some form is present in *C. gabonicum*. The species would certainly be an ideal species for field studies focusing on breeding system and reproductive ecology.

#### Additional specimens examined

**(paratypes). GABON**: Ogooé-Maritime, Toucan, 1°47'S, 9°53'E, 29 May 2002 (fl bud), *H.P. Bourobou Bourobou, G. Niang-Essouma & T. Nzabi 623* (K, MO, P, WAG); 50 km SE of Lambaréné, 1°4'S, 10°30'E, 30 September 1968 (fl), *F.J. Breteler 5747* (BR, K, MO, WAG); Rabi, 1°55'S, 9°50'E, 24 March 1990 (st), *J. Breteler & C.C.H. Jongkind, J. Wieringa & J.M. Moussavou 9437* (BR, WAG); about 30 km E of Lastoursville, 0°40'S, 13°00'E, 20 November 1991 (fl bud), *F. J. Breteler & C.C.H. Jongkind 10609* (WAG); 5–30 km NNW of Ndjolé, 0°5'S, 10°45'E, 21 April 1992 (f bud, fr), *F.J. Breteler, C.C.H Jongkind. & J. Wieringa 10979* (WAG); Moyen-Ogooué, ca. 20–30 km NNW of Ndjolé, 0°3'S, 10°45'E, 1 October 1994 (fl), *F.J. Breteler, B.J.M. Breteler & Klein Breteler 13110* (WAG); Ogooué-Maritime, Rabi-Kounga, route Divangui, 1°54'S, 9°46'E, 14 July 1998 (fl bud), *F.J. Breteler, J.M. Moussavou, J. Nang & O. Pascal 14428* (WAG); Rabi 51, 1°55'S, 9°53'E, 1 March 2007 (st), *J. Choo 1042* (BR); about 30 km NW of Doussala, in the direction of Bongo, 2°38'S, 11°38'E, 16 March 1988 (fl), *J.J.F.E. De Wilde & C.C.H. Jongkind 9392* (BR, K, MO, WAG); Abanga, chantier C.E.T.A. 0°12'S, 10°11'E, 3 June 1963 (st), *N. Hallé 2170* (P); Moyen-Ogooué, Camp Mboumi, 0°25'S, 10°50'E, 1 September 1999 (fl), *Y. Issembe 244* (WAG); Concession Murel & Prom près du Lac Ezanga, 1°5'S, 10°13'E, 51 m, 24 November 2013 (st), *O. Lachenaud, D. Ikabanga, E. Akouangou, J.Y. Serein, E. Bidault, Y. Issembe, A. Boupoya & J.D.D. Kaparadi 1609* (BRLU); 2 km SE of Forestry Camp Waka, situated ca. 32 km SE of Sindara, Waka River basin, 1°14'S, 10°53'E, 10 December 1983 (fr), *A.M. Louis, F. Breteler & J. De Bruijn 1248* (WAG); Rabi (parcelle Smithsonian) code dans la parcelle: CRATGF, 1°55'S, 9°52'E, 30m, 18 March 2011 (st), *D. Nguema, H. Memiague, P. Bissiemou, E. Mounoumou, G. Moussavou, L. Tchignoumba, D. Bikissa & M.W. Mbanding 1311* (BRLU); chantier CEB, ca. 50 km SW of Doussala, 2°36'S, 10°35'E, 21 August 1985 (fl, fr), *J.M. Reitsma & B. Reitsma 1342* (BR, WAG); at logging site of CBG, ca. 5 km beyond checkpoint Divangui, 1°50'S, 10°0'E, 29 October 1990 (fr), *I. Van Nek 152* (WAG); Nyanga, Moukalaba Doudou National Park, 2°44'S, 10°30'E, 20 February 2004 (fl, fr), *J. van Valkenburg, L. Ngok Banak, Y. Issembé & T. Nzabi 2872* (BR, K, WAG); Moyen-Ogooué, Ezanga, Layon D ouest, 1°5'S, 10°14'E, sd (fl) *C.M. Wilks 2466* (WAG).

### Identification key of the species of *Craterispermum* present in the Lower Guinean and Congolian Domains

**Table:** 

1	Bracteoles 3–6 mm long, long aristate; peduncles 1–5 mm long	**2**
–	Bracteoles shorter, 0.3–2(–6) mm long, broadly triangular, ovate or subtruncate; peduncles 1.4–23 mm long	**3**
2	Stipules 5–11 mm long, with short and broadly triangular tips, 1–3(–4.5) mm long; 5–6 pairs of secondary veins; flowers 5-merous; calyx lobes equal; tertiary and higher order venation laxly and irregularly reticulate; leaf blades 11–25.5 × 4–8 cm	***C. aristatum* Wernham** (SW Cameroon, SE Nigeria)
–	Stipules 5–16 mm long, with long and narrowly triangular tips, 4–13 mm long; 10–12 pairs of secondary veins; flowers 4-merous; calyx lobes unequal; tertiary and higher order venation closely and more or less regularly reticulate; leaf blades 6.7–14 × 2–4.8 cm	***C. sonkeanum* Taedoumg & Hamon** (Equatorial Guinea, Gabon)
3	Tertiary and especially quaternary venation obscure on both surfaces and/or very lax in fresh condition; leaf blades always coriaceous	**4**
–	Tertiary and quaternary venation conspicuous on both surfaces; leaf blades coriaceous or papyraceous	**8**
4	Twigs decurrently ridged, peduncles often slender, 4–150 mm long, erect or curved, fruits red at maturity ***C. inquisitorium* Wernh** (Cabinda, Congo, Democratic Republic of the Congo, Gabon)
–	Twigs not decurrently ridged, peduncles stout, (0.6–)2–23 mm long, always erect, fruits violet or dark blue to black at maturity	**5**
5	Inflorescences subcapitate and completely covered by imbricate outer bracts (somewhat resembling an immature fruit) when young, secondary nerves clearly ascending and forming acute angles with the midrib; bracts overlapping one another at least at the base; bracts and bracteoles 6–8 × 5–8 mm and 6 × 3 mm respectively in brevistylous morph and ca. 2 × 1.5–2 mm and 0.7–2 × 1–2 mm respectively in longistylous morph; corolla tube 6–12 mm and 4–5 mm long for brevistylous and longistylous flowers respectively	***C. gabonicum* Taedoumg & De Block** (Gabon)
–	Inflorescences mostly branched and not completely covered by the outer bracts when young, secondary nerves more or less perpendicular to the midrib; bracts not overlapping one another; bracts and bracteoles ca. 1–4 and ca. 1–2 mm long respectively, not differing between morphs; corolla tube 4–8.5 long mm in both morphs	**6**
6	Bracts and bracteoles ca. 4 mm and ca. 2 mm long, respectively; inflorescences 6–90 mm long, moderately to very compact, subcapitate or consisting of 2 branches, each up to 60 mm long; peduncle 1.1–26 mm long; leaf blades 7–35 × 2.5–13.5 cm; corolla tube 6–8.5 mm long; calyx tube 1–1.3 mm long	***C. ledermannii* K.Krause** (Cameroon, Equatorial Guinea, Gabon)
–	Bracts and bracteoles more or less equal, 1–1.5 mm long; inflorescences 2.2–20 mm long, very compact, capitate, subcapitate or consisting of 2–3 branches, each 4.5–15 mm long; peduncle (0.6–)2–7 mm long; leaf blades 6–23 × 1.5–8 cm; corolla tube ca. 4 mm long; calyx tube 0.4–0.7 mm long	**7**
7	Inflorescences consisting of 2–3 branches or subcapitate, each 4.5–15 mm long; fruits urceolate to subglobose at maturity, usually wider at the base than at the tip; leaf blades generally not glossy above; young twigs often smooth, always cylindrical; fruits sessile or very rarely shortly pedicellate (pedicels ca. 2 mm)	***C. robbrechtianum* Taedoumg & Sonké** (Cameroon, Gabon)
–	Inflorescences capitate; fruits ovoid at maturity; leaf blades usually glossy above; young twigs often granular in outlook; often quadrangular and canaliculate near nodes; fruits sessile	***C. capitatum* Taedoumg & De Block** (SW Cameroon, Congo, Democratic Republic of the Congo, SE Nigeria)
8	Stipules with conspicuous narrowly triangular tips, tip 1.5–8 mm long; fruits pedicellate; venation more or less regularly reticulate with secondary veins parallel between them and more or less perpendicular to midrib	**9**
–	Stipules with more ovate and short tips, tip not exceeding ca. 1 mm long; fruits sessile; venation irregularly reticulate with secondary veins not perpendicular to midrib	**11**
9	Stipules persistent; leaf blades papyraceous, 3.3–11 × 0.9–3.5 cm; fruits shortly pedicellate, pedicels 1–1.5 mm long; inflorescences with 1–4 flowers; peduncles 0.5–4.5 mm long; inter-secondary and tertiary venation parallel and more or less perpendicular to midrib	***C. parvifolium* Taedoumg & Sonké** (Cameroon, Equatorial Guinea, Gabon)
–	Stipules caducous; leaf blades coriaceous or subcoriaceous, 5–25 × 1.9–7.5 cm; fruits long pedicellate, pedicels 2.5–9 mm long; inflorescences 3- to several-flowered; peduncles 0.5–9 mm long; intersecondary and tertiary venation not as above	**10**
10	Twigs decurrently ridged but otherwise smooth; 6–12 pairs of secondary veins, leaf blades 5–15 × 1.7–5.3 cm; peduncles 4–9 mm long	***C. caudatum* Hutch**
	(Cameroon, Gabon, Guinea Conakry, Ghana, Ivory Coast, Nigeria, Senegal)
–	Twigs not decurrently ridged with surface granular; 14–16 pairs of secondary veins; leaf blades 8.5–25 × 3.2–7.5 cm; peduncles 0.5–5 mm long	***C. deblockianum* Taedoumg & Hamon** (Gabon)
11	Stipules caducous; inflorescences sessile, subcapitate; leaves subcoriaceous, margins revolute when dry; tertiary and higher order venation closely reticulate; young twigs with bark quickly woody, folded longitudinally and more or less corky in dry condition	***C. rumpianum* Taedoumg & Hamon** (SW Cameroon)
–	Stipules persistent; inflorescences pedunculate, branched or subcapitate especially in young stage; leaves coriaceous or papyraceous, margins not revolute when dry; tertiary and higher order venation closely or laxly reticulate; young twigs not as above	**12**
12	Peduncles (7–)10–20 mm long, slender, leaf blades papyraceous to rarely subcoriaceous, tertiary venation and higher laxly reticulate; inflorescences 2–3-branched, rarely subcapitate especially at young stage; bracteoles and flowers laxly placed; branches (1.5–)4.5–21 mm long; acumen 7–18 mm long	***C. cerinanthum* Hiern** (Cameroon, Congo, DR Congo, Gabon, Equatorial Guinea (Annobon), Nigeria, Principe, Sao Tomé)
–	Peduncles 1.4–7(–10) mm long; stout, leaf blades subcoriaceous to coriaceous; tertiary venation and higher densely reticulate; inflorescences subcapitate, rarely shortly 2-branched [<4(4.9) mm long each]; bracteoles and flowers very congested; acumen 5–12.5 mm long	***C. schweinfurthii* Hiern** (Angola, Burundi, Cameroon, Central African Republic, Chad, Congo, Democratic Republic of the Congo, Ethiopia, Gabon, Kenya, Malawi, Mozambique, Nigeria, Rwanda, Sudan, Tanzania, Uganda, Zambia, Zimbabwe)

## Supplementary Material

XML Treatment for Craterispermum capitatum

XML Treatment for Craterispermum gabonicum
